# Differential inhibition of activity, activation and gene expression of MMP-9 in THP-1 cells by azithromycin and minocycline versus bortezomib: A comparative study

**DOI:** 10.1371/journal.pone.0174853

**Published:** 2017-04-03

**Authors:** Jennifer Vandooren, Sofie Knoops, João L. Aldinucci Buzzo, Lise Boon, Erik Martens, Ghislain Opdenakker, Elzbieta Kolaczkowska

**Affiliations:** 1 Laboratory of Immunobiology, Rega Institute for Medical Research, University of Leuven, KU Leuven, Leuven, Belgium; 2 Department of Evolutionary Immunology, Jagiellonian University, Krakow, Poland; Uniwersytet Gdanski, POLAND

## Abstract

Gelatinase B or matrix metalloproteinase-9 (MMP-9) (EC 3.4.24.35) is increased in inflammatory processes and cancer, and is associated with disease progression. In part, this is due to MMP-9-mediated degradation of extracellular matrix, facilitating influx of leukocytes into inflamed tissues and invasion or metastasis of cancer cells. MMP-9 is produced as proMMP-9 and its propeptide is subsequently removed by other proteases to generate proteolytically active MMP-9. The significance of MMP-9 in pathologies triggered the development of specific inhibitors of this protease. However, clinical trials with synthetic inhibitors of MMPs in the fight against cancer were disappointing. Reports on active compounds which inhibit MMP-9 should be carefully examined in this regard. In a considerable set of recent publications, two antibiotics (minocycline and azythromycin) and the proteasome inhibitor bortezomib, used in cancers, were reported to inhibit MMP-9 at different stages of its expression, activation or activity. The current study was undertaken to compare and to verify the impact of these compounds on MMP-9. With exception of minocycline at high concentrations (>100 μM), the compounds did not affect processing of proMMP-9 into MMP-9, nor did they affect direct MMP-9 gelatinolytic activity. In contrast, azithromycin specifically reduced MMP-9 mRNA and protein levels without affecting NF-κB in endotoxin-challenged monocytic THP-1 cells. Bortezomib, although being highly toxic, had no MMP-9-specific effects but significantly upregulated cyclooxygenase-2 (COX-2) activity and PGE_2_ levels. Overall, our study clarified that azithromycin decreased the levels of MMP-9 by reduction of gene and protein expression while minocycline inhibits proteolytic activity at high concentrations.

## Introduction

**Fig 1:**
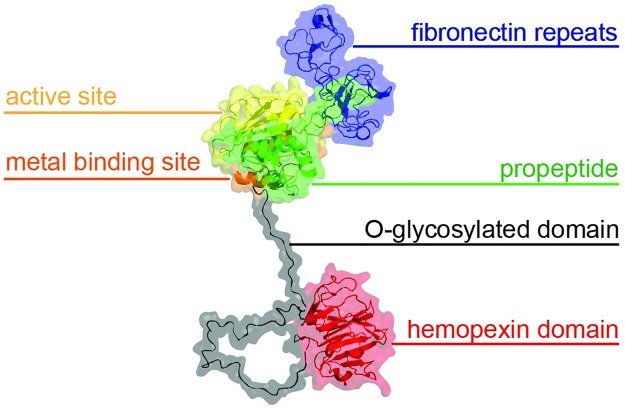
Multi-domain structure of proMMP-9. 3D molecular structure of the full-length human proMMP-9 monomer. The catalytic site is formed by the Zn^2+^ -binding domain (orange) and the active site (yellow) and is highly conserved within the MMP family. MMPs are secreted as pro-enzymes, containing a propeptide domain (green) which interacts with the catalytic Zn^2+^ ion, thereby keeping the enzyme inactive. The fibronectin repeats (blue) are only present in gelatinases (MMP-2 and MMP-9) and assist the catalysis of large substrates such as gelatins. The O-glycosylated domain (black) is a unique domain that, as its name suggests, is flexible and heavily glycosylated. This domain lends the MMP-9 molecule a high degree of flexibility and the ability to reach cleavage sites on long substrates. Finally, the hemopexin domain (red) is present in several MMPs and has a range of functions including substrate binding, inhibitor binding and binding to cell surface receptors [13, 14].

Proteolysis of extracellular matrix (ECM) is an important aspect of inflammatory reactions [1]. Whereas ECM remodeling is necessary to enable leukocytes to enter sites of infection to eliminate micro-organisms, exaggerated modifications may lead to tissue destruction and bone and cartilage deformations, such as those observed in rheumatoid arthritis [2]. Therefore, matrix remodeling is under tight control by protease inhibitors. A known class of matrix remodeling enzymes are the matrix metalloproteinases (MMPs) which, as their name suggests, faithfully rely on the presence of a catalytic metal-ion (Zn^2+^) for their activity [3]. These enzymes are inhibited by endogenous inhibitors, named tissue inhibitors of metalloproteinases (TIMPs). Additionally, all MMPs are secreted with a propeptide domain (Fig 1) which interacts with the catalytic zinc-ion and also functions as an inhibitor of enzyme activity, keeping the pro-enzyme (e.g. proMMP-9) catalytically inactive [4]. Upon the step-wise removal of this propeptide, for example by MMP-3, MMPs become fully activated and capable of degrading their substrates [5]. Among MMPs, MMP-9 is unique from several points of view. Structurally, MMP-9 has an additional domain, called the O-glycosylated domain which offers this MMP extreme flexibility and allows the protease to ‘crawl’ along large substrates [6]. In addition, we recently discovered that this flexibility allows the enzyme to bend into doughnut-shaped homotrimers which differentially interact with TIMP-1 during angiogenesis [7]. From the physiological perspective, MMP-9 is uniquely placed as it is abundantly secreted by neutrophils which, in contrast to other cell types, pre-store MMP-9 in secretory granules, ready for fast release [8]. As a consequence, MMP-9 is associated with many acute and chronic inflammatory diseases: from acute inflammation and shock syndromes to autoimmune diseases and cancer [9, 10]. Moreover, the role of MMP-9 in inflammation was shown to go beyond leukocyte recruitment and also to affect apoptosis [11] and expression of other enzymes such as cyclooxygenase [12]. MMP-9 may be inhibited in several ways. The conversion of the inactive pro-enzyme into its activated form may be blocked, the activated enzymes may be directly inhibited and the production of proMMP-9 may be blocked at the transcriptional level. Once formed, the enzyme mRNA may even be silenced [13].

For decades, major investments have been done by the pharmaceutical industry to generate drugs inhibiting MMPs. Whereas this was primarily with the aim to block tumor cell invasion and metastasis, more recently, it has been recognized that such pharmaceuticals may have a future in inflammation research and therapy [15, 16]. From gene knockout studies in mice, it has been deduced that MMP-9 inhibition may be useful in chronic organ-specific auto-immune diseases such as multiple sclerosis [17], diabetes [18] and rheumatoid arthritis [19], whereas the use of MMP-9 inhibitors needs to be questioned for the treatment of systemic auto-immune diseases [20]. In addition, recent studies indicate that MMP-9 inhibition may also be beneficial in acute infections and inflammatory syndromes, including endotoxinemia [21, 22]. However, not many practical MMP inhibitors exist and the ones available are not therapeutically useful due to a lack of specificity and off-target effects. A persistent lack of knowledge on the complexity of MMP biology hampers the development of safe and effective MMP-targeting drugs [15, 16].

For the above reasons, the search for MMP inhibitors continues. One of the possible approaches to achieve the above goal is to scrutinize active compounds which are already used clinically for other purposes, for their possible impact on MMPs. During the past years, three of such compounds were frequently reported to yield downregulated expression of gene or protein levels of (pro)MMP-9, or alternatively, to inhibit activation of proMMP-9 or activity of MMP-9. However, in the case of MMPs which are released in inactive form and then become activated, there is a common misinterpretation of methodological approaches to qualitatively verify the presence of each form. This especially concerns gelatin gel zymography which is a technique that is commonly used to detect gelatinases (in particular MMP-2 and MMP-9) in complex biological samples (e.g. plasma and tissue extracts). This technique exploits the fact that MMPs, after electrophoretical separation (SDS-PAGE), can be refolded into active enzymes, including proMMPs and MMPs that were previously attached to inhibitors. Therefore, this technique gives information on the potential gelatinolytic activity present in each sample but yields no information about the net proteolytic activity, a commonly made error in interpretation [23].

Many publications on MMP-9 inhibitors report that minocycline, azithromycin and bortezomib possess one or several of the above capacities (S1 Table). For example, human monocytes and PBMCs treated with azithromycin produced less MMP-9 protein [24] as did airways of lung transplant patients treated with azithromycin [25]. Azithromycin is commonly used as an antibiotic in the treatment of respiratory, urogenital and dermal infections. Besides antibiotic properties it also has immunomodulatory properties, and is therefore also being used for the treatment of chronic inflammations such as panbronchiolitis, post-transplant bronchiolitis and rosacea. However, a clear-cut mechanism of this anti-inflammatory effect is still not known [26]. Minocycline is a broad-spectrum tetracycline antibiotic, mainly used for treatment of acne vulgaris and sexually transmitted diseases. While its anti-microbial activities are based on inhibition of the bacterial 30S ribosomal subunit and inhibition of protein synthesis, it is believed that the non-microbial activities are due to the inhibition of several enzymes, including MMPs [27–29]. Minocycline and azithromycin are used in similar diseases e.g. during infection with *Chlamydia* spp. [30] and are more effective when used together [31]. In contrast, bortezomib is a proteasome inhibitor and thus inhibits intracellular degradation of proteins, leading to cell apoptosis [32]. Since MMP-9 also possesses anti-apoptotic effects through its hemopexin domain [11, 33], bortezomib might also target MMP-9. In the clinic, bortezomib is used as a treatment of multiple myeloma (MM) [34].

Based on the reports on effects of minocycline, azythromycin and bortezomib on MMP-9 we undertook biochemical and immunological studies to complement existing studies and to compare the possible inhibitory mechanisms against MMP-9 and at which stage of its expression or activation these act. Against prevailing notion, we showed that decreased levels of MMP-9, observed in macrophage-like THP-1 cells incubated with bortezomib, resulted entirely from its cytotoxic effect and this drug did not affect the activity of the protease. Conversely, minocycline and azithromycin did not reduce cell viability but mainly prevented MMP-9 expression at the gene and/or protein levels. However, these two antibiotics did not have an effect on activation of proMMP-9 and only high doses of minocycline (50–100 μM) affected gelatinolytic activity.

## Materials and methods

### Reagents

Bortezomib (sc-217786), azithromycin (sc-254949), minocycline (sc-203339) and SB-3CT (sc-205847) were purchased from Santa Cruz Biotechnology (TX, USA). LPS from *Escherichia coli* 0111:B4 (L4391) was purchased from Sigma-Aldrich (St. Louis, MO, USA). Recombinant human full-length proMMP-9 (92 kDa) was expressed in Sf9 insect cells and purified by gelatin-Sepharose chromatography. ProMMP-9 was activated by incubation with the catalytic domain of stromelysin-1/MMP-3 (cat. No. 444217, Merck Millipore, Darmstadt, Germany). The recombinant expression, purification and activation of proMMP-9 were performed as described previously [14, 35].

### Gelatin zymography

Samples in non-reducing loading buffer were loaded on standard 7.5% polyacrylamide gels containing 0.1% gelatin. After electrophoresis the gels were washed twice for 20 minutes with 2.5% Triton X-100 to remove SDS. Next the gels were incubated overnight at 37°C in incubation buffer (50 mM Tris, 10 mM CaCl_2_, 0.02% NaN_3_, 1% Triton X-100, pH 7.5) for gelatin degradation. For activity inhibition experiments, the gel was sliced into separate lanes and each lane was treated separately from this point on. Specifically, for these experiments, all lanes in the gels were loaded with equivalent quantities of human recombinant MMP-9 and after electrophoresis these gels were sliced into strips. Next, different concentrations of compounds were added to the incubation buffer and each strip of gel was incubated with a different compound/concentration. Finally, the gels were stained with the PhastGel Blue R-350 staining kit (GE Healthcare, Piscataway, NJ, USA) and the densities of the bands were analyzed with the ImageQuant TL software (GE Healthcare, Piscataway, NJ, USA)[23].

### Gelatin degradation assay

A gelatin degradation assay [36] was used to study the inhibitory effect of bortezomib, azithromycin, minocycline and SB-3CT on the degradation of gelatin by MMP-9. Briefly, 10 nM of active MMP-9 was incubated with 60 μM, 40 μM and 20 μM of the test compound and incubated for 30 min at 37°C. Next, a fluorogenic gelatin substrate (DQ-gelatin^™^, Invitrogen, Carlsbad, CA, USA) was added to this mixture and the increase in fluorescence was recorded every 10 min for 2 hours. Percentages of inhibition were calculated by comparing the initial velocity of each condition with a condition without compound.

### Cell culture, viability and experimental design

THP-1 cells (10^6^ cells in 1 ml RPMI medium, without antibiotics, growth factors or serum) were seeded in each well of 24-well plates and allowed to settle for 1 hour. Next, 50 μl of the test compound was added followed by 50 μl of LPS (final concentration of 10 μg/ml). After 24h (37°C and 5% CO_2_) the supernatants and cell pellets were collected (1200 rpm, 5 min). THP-1 cell viability was tested using 3-(4,5-dimethylthiazol-2-yl)-2,5-diphenyltetrazolium bromide (MTT). MTT was purchased (cat. No. M5655, Sigma-Aldrich, St. Louis, MO, USA) and dissolved at 2 mg/ml in PBS and aliquots were stored at -20°C. The MTT assay was performed according to the suppliers instructions. Absorbance of MTT converted into formazan was measured at a wavelength of 570 nm with background subtraction at 630 nm. The obtained data was presented as percentage of the LPS condition. For cell culture medium samples, the total protein content was determined and used to normalize sample preparation before gelatin zymography. HUVEC cell culture and experimental procedures can be found as supplementary protocol 1 (S1 Protocol)

### RNA extraction and qPCR

Total RNA was extracted using the RNeasy Mini Kit (74106, Qiagen, Hilden, Germany). Next, the quality and concentration of the extracted RNA was determined with the use of a NanoDrop Spectrophotometer and, subsequently, complementary DNA was generated using the High-Capacity cDNA Reverse Transcription Kit (Applied Biosystems). Real-time qPCR was performed by using PrimeTime^®^ Predesigned qPCR Assays, purchased from Integrated DNA Technologies (IDT, Coralville, IA, USA). The following gene expression assays were used: *MMP2*, Hs.PT.58.39034246; *MMP9*, Hs.PT.58.22814824.g; *TIMP1*, Hs.PT.58.27632928; *NFKBIA*, Hs.PT.58.2372284.g; *PTGS2*, Hs.PT.58.77266. Expression levels were normalized to the expression housekeeping gene *GADPH* Hs.PT.39a.22214836.

### ELISA & Western blot

ELISAs were performed on cell culture supernatant of treated THP-1 cells. Levels of IL-1β were determined using a commercially available Human IL-1 beta DuoSet ELISA (cat. No. DY201-05, R&D Systems, Minneapolis, MN, USA). Prostaglandin E_2_ (PGE2) levels were determined by using a PGE2 express ELISA kit (cat. No. 500141, Cayman Chemical Company, Ann Arbor, MI, USA). For Western blot analysis, intracellular proteins from 2x10^6^ treated THP-1 cells were extracted in RIPA buffer (N653, AMRESCO VWR life sciences, Radnor, PA, USA) supplemented with protease inhibitors (Complete mini, EDTA-free tablets, Roche, Basel, Switzerland) and phosphatase inhibitors (phosphatase inhibitor cocktail 2&3, Sigma-Aldrich, St. Louis, MO, USA) using the protocols provided by the suppliers. The total protein concentration of each extract was determined using a BCA protein assay kit (cat. No. 23225, Pierce—Thermo Scientific, Waltham, MA, USA). Next, 20 μg of each cell extract was separated on a mini-gel system (Amersham ECL Gel Box), using a 4–12% precast Amersham ECL Gel (GE Healthcare, Piscataway, NJ, USA) and Tris-glycine electrophoresis buffer, under reducing conditions. Thereafter, proteins were blotted onto a PVDF membrane using a Trans-Blot Turbo transfer system (Bio-rad, Hercules, CA, USA). The following primary antibodies were used: anti-Phospho-NF-κB p65 (Ser536) (Rabbit mAb #3033, Cell Signaling Technology, Danvers, MA, USA), anti-actin (rabbit pAb, 20536-1-AP, protein tech, Rosemont, IL, USA), anti-caspase-4 (rabbit pAb, C4481, Sigma-Aldrich, St. Louis, MO, USA) at dilutions of, respectively, 1/1000, 1/2000 and 1/250.

## Results

### Effects on MMP-9 proteolytic activity—Gelatin degradation

**Fig 2:**
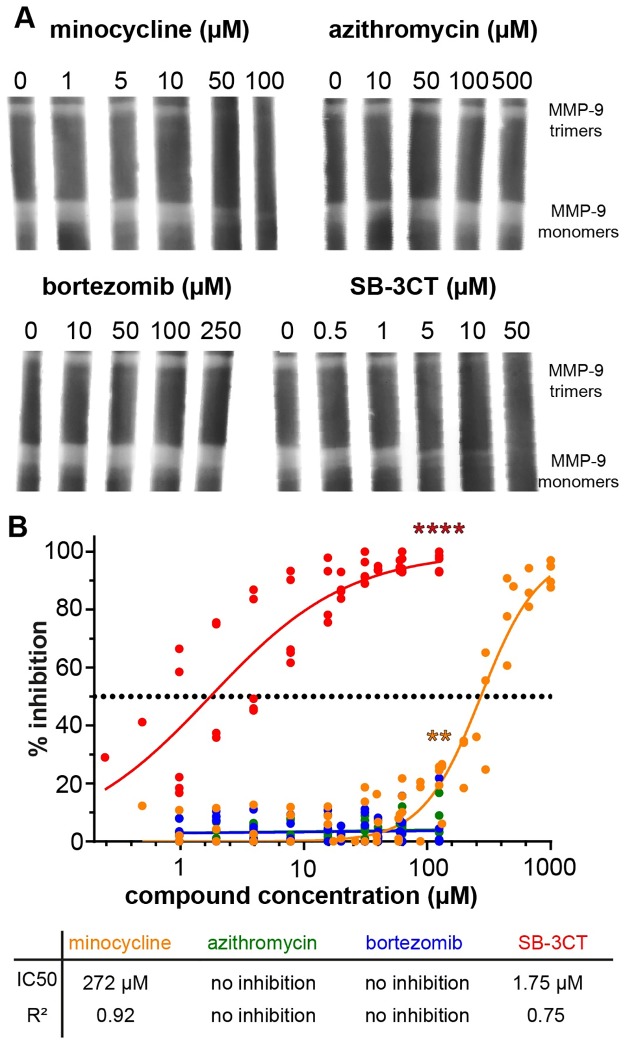
The effect of minocycline, azithromycin, bortezomib and SB-3CT on MMP-9-mediated gelatinolysis. (A) gelatin zymography of MMP-9 mixtures (MMP-9 trimers and MMP-9 monomers). Each gel slice was incubated with a different concentration of each compound, as indicated on top of the lanes. Representative image of two independent experiments. (B) Percentage of inhibition of MMP-9 mediated gelatinolysis, as measured using a gelatin degradation assay. Compound concentration ranged from 1000 μM to 0.24 μM. Data combined from seven independent experiments, including different concentration ranges and a dose-response curve was fitted using non-linear regression. Higher concentrations of bortezomib and azithromycin were not tested in the gelatin degradation assay, due to solvent interference. Individual data points are shown. Statistical analysis was performed for the data at 125 μM by using a Bonferroni's multiple comparison test. **, p ≤ 0.01; **** p ≤ 0.0001.

In order to evaluate effects of the tested compounds on MMP-9 we took the downstream approach i.e. first we investigated whether minocycline, azithromycin or bortezomib could inhibit MMP-9 gelatinolytic activity. To test high concentrations, compounds were added *in vitro* to the developing buffer of a gelatin zymography gel and SB-3CT, a molecule known to inhibit MMP-9 activity, was used as a control (Fig 2A). While SB-3CT inhibited MMP-9 at concentrations lower than 5 μM, bortezomib and azithromycin had no effect on the development of MMP-9 activity. As expected from previous research [27], minocycline inhibited MMP-9 when present at high concentrations (>50 μM). Next, we confirmed these results by using a second technique: a microtiter-based gelatin degradation assay [36] (Fig 2B). With this test, we were able to compare the inhibitory profile of all four compounds in one plate. While bortezomib and azithromycin did not inhibit gelatinolysis, the IC50 of SB-3CT and minocycline were respectively 1.75 μM and 272 μM, which is in agreement with previous research [27]. While SB-3CT showed a strong inhibitory profile, no effect was observed from azithromycin or bortezomib.

### Effects on MMP-9 activation by MMP-3

**Fig 3:**
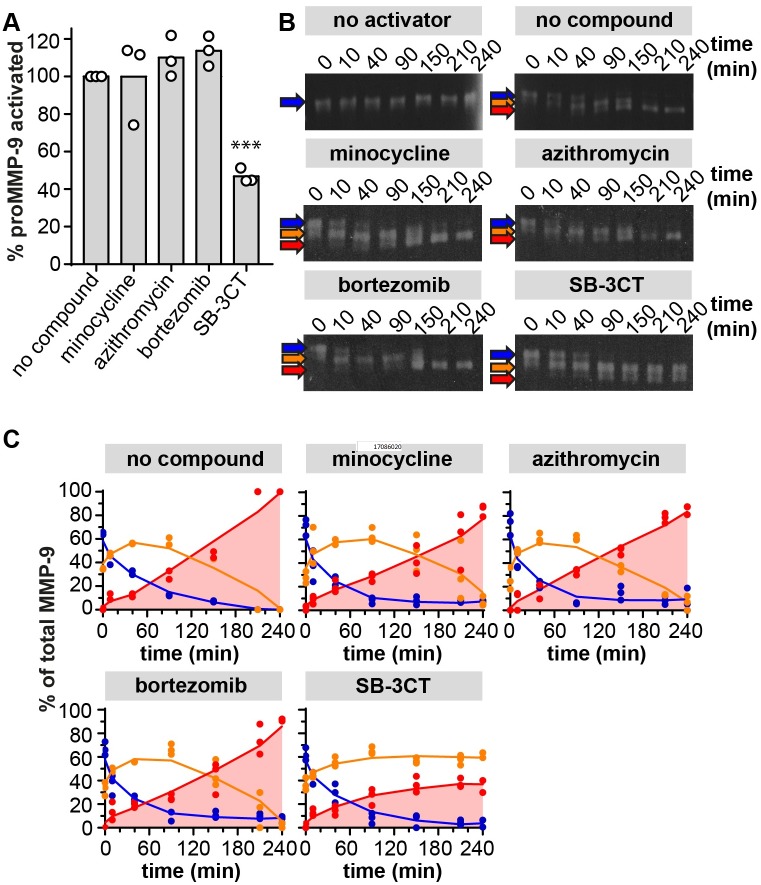
Inhibition of proMMP-9 activation by MMP-3. (A) The influence of minocycline, azithromycin, bortezomib and SB-3CT on the activation of proMMP-9 into MMP-9, measured by assessing the gelatinolytic activity of proMMP-9 after incubation with a proMMP-9 activator (catalytic domain of MMP-3) in the presence of the compounds. Data were compared to a condition without compound and expressed as percentage of activated proMMP-9. Individual data points, each representing a separate experiment, are shown. The bars represent the mean value. Inhibition of proMMP-9 activation by SB-3CT was significantly different as determined with a Bonferroni's multiple comparisons test. ***, P ≤ 0.001. (B) Zymography analysis of proMMP-9 activation by cdMMP-3 in the presence of minocycline, azithromycin, bortezomib and SB-3CT. Zymograms, representative for three experiments, show the stepwise activation of proMMP-9, from the full-length enzyme (proMMP-9, blue), to the partially activated enzyme (proMMP-9’, orange) and the fully activated enzyme (MMP-9, red) as indicated by the arrows. (C) Densitometric analysis of the zymograms. The proportions of pro, intermediate and activated MMP-9 are shown as the percentages of the total MMP-9. Individual data points, each representing a separate experiment, are shown.

MMP-9 is secreted as an inactive pro-enzyme (proMMP-9) which becomes fully active upon removal of the inhibitory pro-domain by other proteinases. So far, the best studied activator of proMMP-9 is MMP-3 which activates proMMP-9 in two steps, each step resulting in a decrease in molecular weight of approximately 5 kDa [37]. Therefore, we tested whether activation by MMP-3 is influenced by the compounds. After two hours of incubating proMMP-9 with MMP-3, no difference in residual MMP-9 gelatinolytic activity was found for minocycline, azithromycin and bortezomib. As expected, SB-3CT generally reduced gelatin degradation (Fig 3A). To study the stepwise activation of proMMP-9 in detail, samples were taken at different times of the activation process, and analyzed by gelatin zymography. A stepwise activation of proMMP-9 (blue, 92kDa) into an intermediate form (orange, 86 kDa) and the fully activated MMP-9 (red, 82 kDa) was also seen in the presence of minocycline, azithromycin and bortezomib (Fig 3B and 3C). Compound SB-3CT delayed the first cleavage into the intermediate proMMP-9 form. Inhibition of MMP-3 activity by SB-3CT at higher concentrations has been reported before and clarifies the delayed activation which we observed here [38].

### Effects on protein expression of (pro)MMP-9

**Fig 4:**
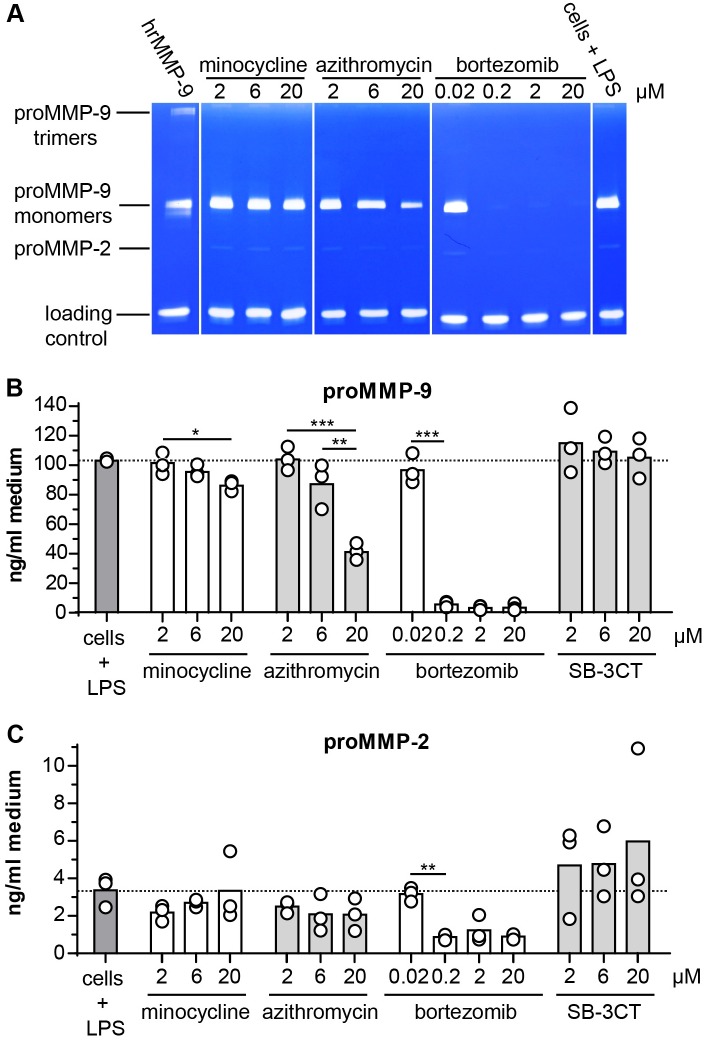
Zymography of THP-1 cells challenged with LPS and treated with minocycline, azithromycin, bortezomib and SB-3CT. (A) Overview image of the zymography analysis showing a compilation of representative zymography gels. Prior to zymography, loading volumes were corrected for the total protein content of each sample. Equal amounts of an internal loading control (±48 kDa MMP-9 mutant lacking the O-glycosylated and hemopexin domain [14]) were included to allow correction for sample processing and loading errors. hrMMP-9, human recombinant proMMP-9. (B) Densitometry analysis of proMMP-9 bands. (C) Densitometry analysis proMMP-2 bands. Individual data points are shown and the bars represent the mean value. Data were statistically analyzed using a Bonferroni's multiple comparison test. *, p ≤ 0.05; **, p ≤ 0.01; *** p ≤ 0.001; **** p ≤ 0.0001; n = 2–3.

**Fig 5:**
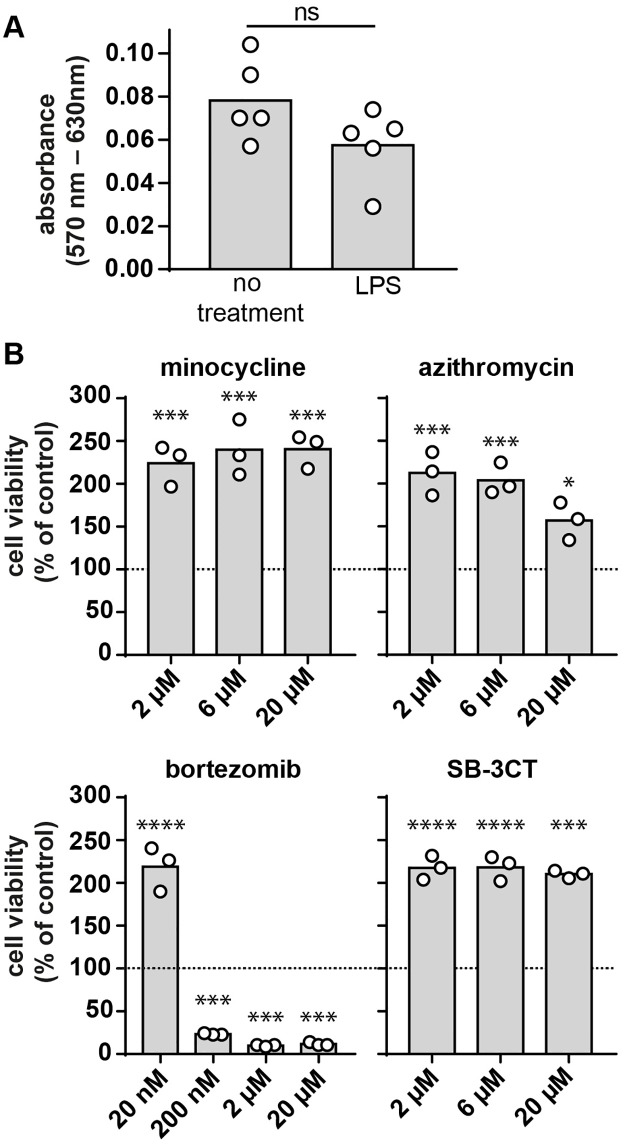
The effect of minocycline, azithromycin, bortezomib and SB-3CT on cell viability of LPS-stimulated THP-1 cells. (A) The effect of LPS on cell viability evaluated by measuring cell metabolic activity (MTT assay). Stimulation of THP-1 cells with LPS had no significant effect on the cell viability as determined with a Mann—Whitney U test. Data represented as background-subtracted absorbance (570 nm– 630nm). Individual data points are shown and the bars represent the mean value. Ns, not significant; n = 5. (B) The effect of LPS stimulation of THP-1 cells in combination with minocycline, azithromycin, bortezomib and SB-3CT on cell viability. The cell viability is expressed as the percentage of cells compared to the condition (LPS only, no compounds). Individual data points are shown and the bars represent the mean value. Mann—Whitney U tests were used to compare with the control condition (LPS condition) *, p ≤ 0.05; **, p ≤ 0.01; *** p ≤ 0.001; **** p ≤ 0.0001; n = 3–5.

The effects of the drugs were subsequently evaluated at the cellular level. MMP-9 is predominantly produced by myeloid cells (e.g. neutrophils, monocytes and macrophages), while the secretion of MMP-9 by other cell types (e.g. fibroblasts, endothelial cells,..) is minimal (S2 Fig). In addition, while neutrophils have MMP-9 pre-stored in secretory granules, mononuclear leukocytes *de novo* synthesize MMP-9 upon stimulation [8]. For these reasons, human myelomonocytic (THP-1) cells are often used to study cellular effects of drugs on MMP-9 secretion. THP-1 cells were incubated with different concentrations of each compound and were then challenged with *E*. *coli* lipopolysaccharide (LPS), mimicking an inflammatory condition [39]. Next, we evaluated the presence of gelatinases by gelatin zymography (Fig 4A). At high concentrations (20 μM), minocycline and azithromycin were able to reduce the amounts of secreted proMMP-9 protein (Fig 4B), while proMMP-2 protein levels remained unaltered (Fig 4C). In contrast, treatment with bortezomib significantly reduced both MMP-2 and MMP-9 levels at concentrations as low as 200 nM. Next, we evaluated the effects of the compounds on cell viability. LPS stimulation did not significantly affect cell viability in the absence of tested compounds (Fig 5A). In contrast, bortezomib treatment resulted in clear cell toxicity at concentrations down to 200 nM in both THP-1 (Fig 5B) cells and HUVEC cells (S1 Fig). Interestingly, the MMP-9 inhibitor SB-3CT (20 μM), minocycline (2 μM) and the antibiotic azithromycin (6 μM) were not cytotoxic but instead stimulated THP-1 cell growth. Low concentrations of bortezomib (20 nM) also stimulated THP-1 cell growth (Fig 5B).

### Effects on MMP-9 gene expression

**Fig 6:**
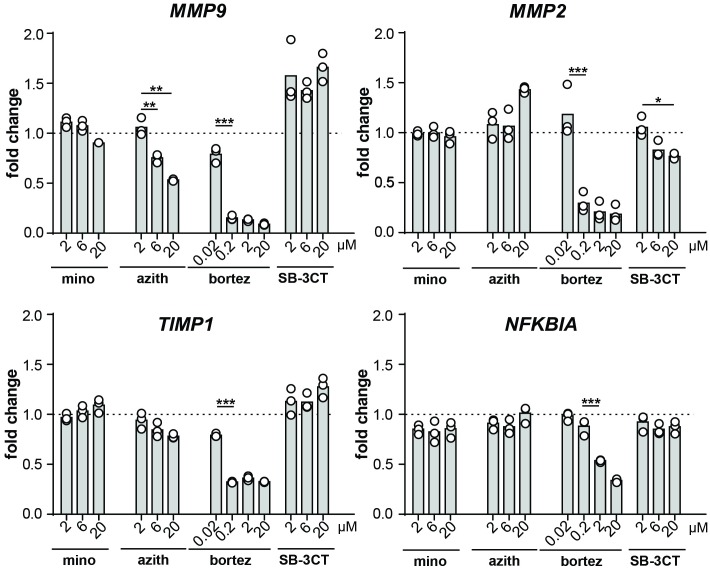
qPCR analysis of LPS-challenged THP-1 cells, treated with minocycline, azithromycin, bortezomib and SB-3CT. Fold changes of *MMP2*, *MMP9*, *TIMP1* & *NFKBIA* mRNA levels are shown, relative to the levels from cells treated with LPS only and corrected towards the housekeeping gene *GADPH*. Individual data points are shown and the bars represent the mean values. Data were statistically analyzed with the use of a Bonferroni's multiple comparison test. *, p ≤ 0.05; **, p ≤ 0.01; *** p ≤ 0.001; n = 3.

Next, we evaluated gene expression of MMP-2 and MMP-9. To avoid effects derived from cell viability, all samples were corrected for total RNA and for the housekeeping gene *GADPH*. At the mRNA level, bortezomib and azithromycin significantly reduced steady-state MMP-9 mRNA levels while minocycline showed a minor but non-significant reduction at 20 μM. Comparable to the protein level, MMP-2 mRNA levels were significantly reduced by bortezomib. (Fig 6). Additionally, we evaluated expression of other genes to verify specificity towards MMP-9. The selected genes were *TIMP1* and *NFKBIA* as the former encodes the endogenous MMP-9 inhibitor TIMP-1 and the latter, NFκB, acts downstream of LPS signaling through Toll-like receptor-4 activation and is a master switch that controls transcription of the majority of pro-inflammatory genes, including MMP-9. In contrast to MMP-9, the expression of *TIMP1* and *NFKBIA* was unchanged by minocycline and azithromycin, strongly suggesting that the effects of azithromycin were MMP-9 specific (Fig 6). In contrast, bortezomib persisted to reduce gene expression by also reducing *TIMP1* and *NFKBIA* mRNA, in line the above mentioned toxicity profile.

### Cell signaling events leading to reduced MMP-9 expression

**Fig 7:**
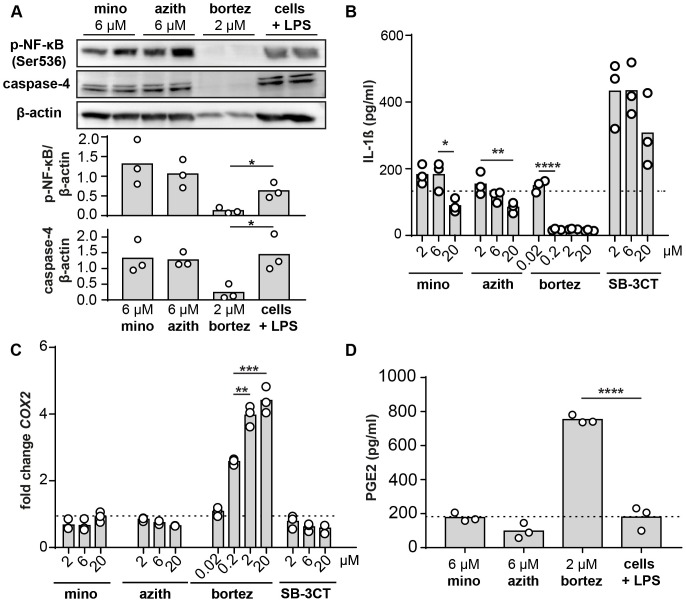
Cell signaling events leading to reduced MMP-9 expression. (A) Western blot analysis of intracellular, phosphorylated NF-κB (Ser536) and caspase-4 in THP-1 cells stimulated with LPS and treated with 6 μM minocycline, 6 μM azithromycin, 2 μM bortezomib or no treatment (cells + LPS without any of the compounds). Western blot images show two samples per condition. Graphs are based on densitometry analysis of Western blot images (n = 3). (B) IL-1β quantification by ELISA. THP-1 cells were challenged with LPS and 20, 6 and 2 μM of minocycline or azithromycin. Bortezomib was used at concentrations of 20 μM, 2 μM, 200 nM and 20 nM. (C) qPCR analysis of THP-1 cells challenged with LPS after treatment with minocycline, azithromycin, bortezomib and SB-3CT. Fold changes *COX2* mRNA levels are shown, relative to cells treated with LPS only and corrected towards the housekeeping gene *GADPH*. (D) PGE_2_ ELISA on cell supernatants of THP-1 cells stimulated with LPS and treated with the compounds. Individual data points are shown and the bars represent the mean value. Horizontal dotted line always indicates cells only treated with LPS. Data were statistically analyzed using a Bonferroni's multiple comparison test. *, p ≤ 0.05; **, p ≤ 0.01; *** p ≤ 0.001; n = 3.

Finally, we investigated which signaling events and pathways drive the specific MMP-9 reduction caused by minocycline and azithromycin. Since MMP-9 is seen as a pro-inflammatory molecule, simultaneously secreted with other molecules (e.g. cytokines) we first evaluated whether NF-κB phosphorylation might be affected. At a concentration of 6 μM neither minocycline nor azithromycin affected NF- κB phosphorylation in comparison to cells only stimulated with LPS (Fig 7A). Among cytokines which can induce MMP-9 expression [39], IL-1β was previously reported to be reduced by azithromycin, independently of NF- κB [40]. Therefore, we next evaluated the presence of IL-1 β in cell supernatants. Indeed, at a concentration of 20 μM a reduction of IL-1β could be found both for azithromycin, but also for minocycline (Fig 7B). In line with all previous results bortezomib in excess of 200 nM resulted in shut-off of IL-1β production, probably by toxicity effects. Interestingly, treatment with SB-3CT resulted in a drastic increase of IL-1β. Previously, the effect of azithromycin on IL-1β was attributed to a reduction of caspase-4, reduced inflammasome activation and IL-1β maturation. Therefore, we also evaluated the intracellular presence of caspase-4 (Fig 7A). Caspase-4 was not altered by minocycline, azithromycin or SB-3CT, while bortezomib again showed a significant decrease. Prostaglandins are mediators of inflammation and apoptotic pathways, which are produced from arachidonic acid by the cyclooxygenase (COX) enzymes [41]. In addition, we have previously shown that MMP-9 deficiency results in augmented PGE-2 secretion [12]. Therefore, we investigated whether the compounds could affect cyclooxygenase-2 (*COX2*) and its product, prostaglandin E2 (PGE2). In contrast, minocycline and azithromycin did not affect *COX2* (Fig 7C). Moreover, these results were complemented with according alterations in secreted prostaglandin E_2_ levels (Fig 7D).

## Discussion

We compared the effects of minocycline, azithromycin and bortezomib on MMP-9 activity, activation and mRNA/protein expression as numerous publications reported that the compounds can affect these processes (for an overview, see S1 Table). In addition, several of these studies yielded different outcomes, did not discriminate between pro or activated forms of MMP-9, did not study activation *versus* activity and lacked information about specific comparisons. Our study revealed that in fact none of these compounds affected activation of the MMP-9 pro-enzyme (for an overview of the results, see Table 1). At high concentrations, minocycline, but not the other two compounds, inhibited MMP-9 proteolytic activity. Previous reports documented an IC50 value of 180 μM for minocycline [27, 42]. Our data indeed show that minocycline inhibits MMP-9 gelatinolytic activity in the micromolar range (IC50 = 272 μM) when tested in our gelatin degradation assay. Peak minocycline concentrations in patients treated with a dose of 200 mg minocycline range between 6 and 19 μM [43]. In this range no inhibition of MMP-9-mediated gelatinolysis was observed which implies that the effect of minocycline on MMP-9 activity felt outside the window for therapeutic treatment. Importantly, we found that azithromycin suppresses MMP-9 mRNA and protein expression by LPS-stimulated human myelomonocytic cells. Pharmacokinetic analysis of azithromycin (500 mg/day) in cystic fibrosis patients revealed sputum concentrations of ±40 μM while blood and serum concentrations were around 1 μM [44]. Therefore, the levels of azithromycin to inhibit MMP-9 expression can be reached in the airways of cystic fibrosis patients treated with azithromycin. Importantly, the effect on MMP-9 gene expression was specific for this MMP as expression of MMP-2 (another gelatinase), TIMP-1 (endogenous MMP-9 inhibitor) and COX-2 (inflammation-activated gene encoding eicosanoids) were not affected by azithromycin and minocycline at these concentrations. Minocycline and azithromycin might therefore be helpful in diseases where MMP-2 and MMP-9 have opposing effects. An example of such a disease is arthritis, where MMP-2 is a disease suppressor whereas MMP-9 contributes to pathology [45].

**Table 1:** Summarizing table of the results.

	minocycline	azithromycin	bortezomib	SB-3CT
**MMP-9 activity**	weak inhibitor	no effect	no effect	strong inhibitor
**MMP-9 activation**	no effect	no effect	no effect	weak effect by inhibiting MMP-3
**MMP-9 mRNA**	trend towards reduction	reduction	reduction	increase
**proMMP-9 protein**	reduction	reduction	reduction	no effect
**MMP-2 mRNA**	no effect	no effect	reduction	reduction
**proMMP-2 protein**	no effect	no effect	reduction	no effect
**IL-1β protein**	reduction	reduction	reduction	increase
**TIMP-1 mRNA**	no effect	no effect	reduction	no effect
**Nfkbia mRNA**	no effect	no effect	reduction	no effect
**Phospho-NF-kB**	no effect	no effect	reduction	no effect
**PTGS2/COX2 mRNA**	no effect	no effect	drastic increase	no effect
**PGE2**	no effect	no effect	drastic increase	no effect

Expression of MMP-9 can be induced by multiple factors such as cytokines, including IL-1β [46]. For this reason we verified if its levels correlated with the effect of the compounds on MMP-9 expression. Indeed, while LPS alone induced IL-1β, this induction was decreased when cells were subsequently treated with minocycline or azithromycin. *De novo* expression of both IL-1β and MMP-9 is regulated by the transcription factor NFκB. However, we showed that (at a 6 μM concentration) the reduction in MMP-9 levels upon treatment with minocycline and azithromycin was independent of NFκB. Specifically, NF-κB-independent reduction of LPS-induced IL-1β secretion was previously described for azithromycin [40] Moreover, treatment of human monocytes with azithromycin had an impaired induction of caspase-4 which was recently described as a new intracellular LPS sensor [47, 48]. Indeed, pharmacokinetics of macrolide antibiotics such as azithromycin show a predominant accumulation in white blood cells [49]. In summary, while we could show an NF-κB-independent effect on IL-1β for azithromycin and minocycline, the effects on caspase-4 were not corroborated in our experiments. However, it is still possible that this caspase-4 dependent effect occurs at higher dosages (> 20 μM).

Whereas the antibiotics minocycline and azithromycin may act directly through effects on MMP-9, effects for bortezomib may be non-specific and predominantly derived from cells under severe stress due to the high toxicity of bortezomib. Peak plasma bortezomib concentrations in patients following a standard drug scheme (1.3 mg/m^2^ on days 1,4,8 and 11 of 21-day cycles) ranged between 0.5 μM and 0.05 μM [50]. This is indeed within our tested concentration range showing high toxicity on THP-1 cells. In addition, we here confirm the double-edged effect of bortezomib, impairing cells at a high concentration (from 100–200 nM) and inducing cell proliferation at a lower concentration (from 10–20 nM), as was previously shown [51]. While our data are in line with the previous report that bortezomib prevents degradation of IκB, and in turn blocks NF-κB activation and suppresses cytokine and survival factor productions [32], we also found that it could drastically induce *PTGS2/COX2* mRNA levels in THP-1 cells. This was further translated to dramatically increased release of PGE_2_, one of the most potent pro-inflammatory prostaglandins [41]. Interestingly, PGE_2_ promotes healing in tissue injuries and elevated levels of PGE_2_ are associated with increased risk of cancer [52]. In particular, cyclooxygenase-2 (COX-2) is often expressed in multiple myeloma (MM) and is a predictor of poor outcome [53] and apoptosis/ treatment resistance. While our results support the combined use of bortezomib and COX-2 inhibitors for the treatment of MM [54], it is feasible for future MM research to investigate if the high *COX2* MM phenotype might be potentiated by bortezomib itself.

The current study was undertaken as a result of critical reading of reports on inhibitory effects of azithromycin, minocycline and bortezomib on MMP-9. Indeed, various overstatements were launched on the basis of clinical studies. First, our findings support the notion that MMPs are regulated at various levels and these need to be carefully assessed when claiming inhibitory effects. Second and foremost, the current study clarified that minocycline and azithromycin can specifically act on MMP-9 expression, but, respectively, less or not on its proteolytic activity or activation by MMPs. Another surprising finding of our work was that bortezomib has limited or no effect on MMP-9 and, besides being highly toxic, drastically induces the secretion of COX-2 which is translated in high amounts of PGE_2_.

## Supporting information

S1 Table Previously described effects of minocycline, azithromycin and bortezomib on MMP-9. **AML**; acute myelogenous leukemia, **BCEC**; bovine corneal endothelial cells, **CoMTb**; conditioned medium from monocytes infected with M. tuberculosis, **COPD**; chronic obstructive pulmonary disease, **DSS**; dextran sodium sulfate, **EAC**; experimental autoimmune carditis, **EAE**; experimental autoimmune encephalomyelitis, **EAN**; Experimental autoimmune neuritis, **ELISA**; enzyme-linked immunosorbent assay, **Fmr**; fragile X mental retardation gene, **HASMCs**; human aortic smooth muscle cells, **HGF**; human gingival fibroblasts, **IHC**; immunohistochemistry, **JEV**; Japanese encephalitis virus, **LPS**; lipopolysaccharide, **MCAO**; middle cerebral artery occlusion, **MM**; multiple myeloma; **MNC**; mononuclear cells, **OGD**; oxygen-glucose deprivation, **PBMC**; peripheral blood mononuclear cells, **PC**; pheochromocytoma, **PVD**; pial vessel disruption, **RPE**; retinal pigment epithelial, **SAH**; subarachnoid hemorrhage, **WB**; western blot, **1**; protein levels determined by ELISA, **2**; protein levels determined by gelatin zymography, **3**; WB protein levels, **4**; IHC protein levels, ↓; decreased, →; no change, ↑; increased.
(DOCX)

S1 Protocol HUVEC culture, viability and experimental design. (DOCX)

S1 Fig The effect of minocycline, azithromycin, bortezomib and SB-3CT on cell viability of LPS-stimulated HUVEC cells. Cell viability is expressed as the percentage of cells compared to the control condition (LPS only, no compounds). Individual data points are shown and the bars represent the mean value. Data were statistically analyzed using a Bonferroni's multiple comparison test. *, p ≤ 0.05; **, p ≤ 0.01; *** p ≤ 0.001; **** p ≤ 0.0001; n = 3.
(DOCX)

S2 Fig Zymography and qPCR analysis of HUVEC cells challenged with LPS and treated with minocycline, azithromycin, bortezomib and SB-3CT. (A) Overview image of a representative zymography gel. To concentrate the sample, cell culture supernatant was first subjected to gelatin affinity chromatography. Loadings are representative for 25 μl cell culture supernatant. MMP-2 appeared as a constitutively expressed gelatinase and no MMP-9 protein was detected. Equal amounts of an internal loading control (±48 kDa MMP-9 mutant lacking the O-glycosylated and hemopexin domains) were included to allow correction for sample processing and loading errors. hrMMP-9, human recombinant proMMP-9. (B) Relative expression of the mRNAs encoded by the human MMP-2, MMP-9 and TIMP-1 genes, as corrected towards that of the housekeeping gene GADPH. While MMP-2 gene expression levels were high and constitutive, MMP-9 expression was insignificant. Compound concentrations were identical to those in panel A. (C) Fold changes of MMP-2 and TIMP-1 mRNA levels, relative to the levels from cells treated with LPS only and corrected towards the housekeeping gene GADPH. Compound concentrations were identical to those in panel A. Individual data points are shown and the bars represent the mean values. Data were statistically analyzed compared to the LPS control using a Bonferroni's multiple comparison test. **, p ≤ 0.01; **** p ≤ 0.0001; n = 3.
(DOCX)
