# Supplementary material for: Differential inhibition of activity, activation and gene expression of MMP-9 in THP-1 cells by azithromycin and minocycline versus bortezomib: A comparative study
Source: PLoS One. 2017 Apr 3;12(4):e0174853. doi: 10.1371/journal.pone.0174853 (PMC5378356; doi:10.1371/journal.pone.0174853)
Supplement: S1 Table — AML; acute myelogenous leukemia, BCEC; bovine corneal endothelial cells, CoMTb; conditioned medium from monocytes infected with M. tuberculosis, COPD; chronic obstructive pulmonary disease, DSS; dextran sodium sulfate, EAC; experimental autoimmune carditis, EAE; experimental autoimmune encephalomyelitis, EAN; Experimental autoimmune neuritis, ELISA; enzyme-linked immunosorbent assay, Fmr; fragile X mental retardation gene, HASMCs; human aortic smooth muscle cells, HGF; human gingival fibroblasts, IHC; immunohistochemistry, JEV; Japanese encephalitis virus, LPS; lipopolysaccharide, MCAO; middle cerebral artery occlusion, MM; multiple myeloma; MNC; mononuclear cells, OGD; oxygen-glucose deprivation, PBMC; peripheral blood mononuclear cells, PC; pheochromocytoma, PVD; pial vessel disruption, RPE; retinal pigment epithelial, SAH; subarachnoid hemorrhage, WB; western blot, 1; protein levels determined by ELISA, 2; protein levels determined by gelatin zymography, 3; WB protein levels, 4; IHC protein levels, ↓; decreased, →; no change, ↑; increased. (DOCX) [file pone.0174853.s001.docx]

**Table S1:** Previously described effects of minocycline, azithromycin and bortezomib on MMP-9

| **Pathology** | **model** | | **Conc. tested** | **Effect on MMP-9** | | | **Comments** | **References** |
| --- | --- | --- | --- | --- | --- | --- | --- | --- |
|  |  | |  | **mRNA expression** | **Protein levels** | **activity** |  |  |
| **AZITHROMYCIN** | | | | | | | | |
| Inflammation | *In vitro* | Human gingival cells | 0.1 - 10 µg/ml |  | ^2^🡪 |  | cultured with serum | [1] |
|  |  | Human osteoclasts | 20 µg/ml | 🡩🡫 culture substrate dependent |  |  |  | [2] |
|  |  | Human monocytes and PBMCs | 33-333 µM |  | ^2^🡫 |  |  | [3] |
|  |  | Co-culture mouse fibroblasts and macrophages | 30 µM |  | ^1^🡫 |  | cultured with serum | [4] |
|  |  | Human bronchial epithelial cells | 30 µg/ml | 🡫, 🡪 | ^1^🡪 |  |  | [5, 6] |
|  | *In vivo* | Human allograft dysfunction |  |  | ^1,2^🡫  Activated MMP-9 levels^2^🡫  MMP-9/NGAL levels^2^🡫 |  |  | [7] |
|  |  | Sputum COPD patients | 250 mg/day 12 months |  |  | 🡪 |  | [8] |
|  |  | Mouse *P. Aeruginosa* pneumonia, lavage fluids | 0.16 g/kg daily |  | ^1^🡩🡫 |  |  | [4] |
|  |  | Rat acute conjunctivitis | 15 mg/g topical ocular | 🡩 |  |  |  | [9] |
| **BORTEZOMIB** | | | | | | | | |
| Cancer | *In vitro* | Human bladder cells | 1 µM |  | ^1^🡫 |  |  | [10] |
|  |  | Human AML cells | 25 nM |  | ^1^🡪 |  |  | [11] |
|  |  | myeloma cells and MNC from BM of MM patients | 20 - 25 nM |  | ^2,3^🡫  Activation🡫 |  | 10% cell toxicity | [12, 13] |
|  | *In vivo* | Orthotopic tumors of 253JB-V human bladder cells | 1 mg/kg |  | ^4^🡫 |  |  | [10] |
| Inflammation | *In vivo* | rat liver I/R injury | 0.1 mg/kg | Ratio MMP-9/MMP-2 gene expression🡩 | Activated MMP-9 ^2^🡫 | 🡫 | Activated MMP-2 levels^2^🡫  MMP-2 activity🡫 | [14] |
| **MINOCYCLINE** | | | | | | | | |
| Inflammation | *In vitro* | Mouse peritoneal macrophages |  |  | ^2^🡫 |  |  | [15] |
|  |  | VEGF stimulated HASMCs | 13-30 µM | 🡫 | ^2^🡫 |  | MMP-2 mRNA 🡪, protein ^2^🡫  TIMP-1 mRNA 🡪 | [16] |
|  |  | Recombinant MMP-9 |  |  |  | IC_50_ = 180 µM | IC_50_ MMP-7 = 125 µM | [17] |
|  |  | TNF-α activated THP-1 cells | 1 nM | 🡫 |  |  |  | [18] |
|  |  | RPE cells | 25 µM | 🡩 |  |  |  | [19] |
|  |  | Rat PC12 cells subjected to OGD | 200 nM |  | ^2^🡫 |  |  | [20] |
|  |  | Human T-cells | 250 µg/ml |  | ^3^🡫 |  |  | [21] |
|  | *In vivo* | Mouse/Rat TBI model | 90 mg/kg,  1 mg/kg (i.v.) | 🡫 | ^2,3^🡫 |  | MMP-2 mRNA and protein ^2,3^🡫 | [22-24] |
|  |  | Mouse DSS-induced colitis | 30 mg/kg/day | 🡫 |  |  |  | [25] |
|  |  | LPS induced neuroinflammation | 150 mg/kg |  | ^2^🡫 |  |  | [26] |
|  |  | MCAO in rats | 2x 30 mg/kg/day |  | ^2^🡫 |  |  | [27] |
|  |  | Rat nerve graft model | 50 mg/kg/day (i.p.) | 🡫 | ^3^🡫 |  |  | [28] |
|  |  | JEV infected mice | 2 x 45 mg/kg/day |  | ^2^🡫 |  |  | [29] |
|  |  | ischaemia in rats/ mice | 50 mg/kg/day  45 mg/kg 2x per day  60 mg/kg/12h  2.5-5 mg/kg/day |  | ^2,3,4^🡫 |  |  | [17, 30-35] |
|  |  | Rabbit atherosclerosis | 1.5/kg/day – 3 mg/kg/day |  | ^1,4^🡫 | 🡫 total MMP |  | [18] |
|  |  | Thromboembolic stroke model in mice | 6 mg/kg i.v. |  | ^2,3^🡫 |  |  | [36] |
|  |  | Rats with SAH | 1 mg/ kg/24h |  | ^3^🡫 |  |  | [37] |
|  |  | VEGF hyperstimulation | 1-100 mg/kg/day (oral) | 🡫 | ^2^🡫 |  |  | [38] |
|  |  | Mouse EAE model | 25 mg/kg/day | 🡫 |  |  |  | [21] |
|  |  | Rat EAC model | 50 mg/kg/day | 🡫 | ^2^🡫 |  |  | [39] |
|  |  | Rat EAN model | 50 mg/kg/day | 🡫 | ^4^🡫 |  |  | [40] |
|  |  | Rat PVD model for small vessel-stroke | 45 mg/kg/day |  | ^3,4^🡫 |  |  | [41] |
| other | *In vivo* | Fragile X syndrome patients (plasma) |  |  | ^2^🡫 |  |  | [42] |
|  |  | *Fmr1* KO mice | 30 mg/kg/day |  | ^2,3^🡫 |  |  | [43] |
|  |  | Rat Alzheimer disease | 50 mg/kg/day (i.p.) |  | ^2^🡫 |  |  | [44] |

**AML;** acute myelogenous leukemia, **BCEC**; bovine corneal endothelial cells, **CoMTb;** conditioned medium from monocytes infected with *M. tuberculosis*, **COPD;** chronic obstructive pulmonary disease, **DSS**; dextran sodium sulfate, **EAC**; experimental autoimmune carditis, **EAE**; experimental autoimmune encephalomyelitis, **EAN**; Experimental autoimmune neuritis, **ELISA;** enzyme-linked immunosorbent assay, **Fmr**; fragile X mental retardation gene, HASMCs**;** human aortic smooth muscle cells**, HGF;** human gingival fibroblasts, **IHC;** immunohistochemistry, **JEV**; Japanese encephalitis virus, **LPS**; lipopolysaccharide, **MCAO**; middle cerebral artery occlusion, **MM;** multiple myeloma; **MNC;** mononuclear cells, **OGD**; oxygen-glucose deprivation, **PBMC;** peripheral blood mononuclear cells, **PC**; pheochromocytoma, **PVD**; pial vessel disruption, **RPE**; retinal pigment epithelial, **SAH;** subarachnoid hemorrhage, **WB;** western blot**, 1;** protein levels determined by ELISA, **2;** protein levels determined by gelatin zymography, **3;** WB protein levels, **4;** IHC protein levels, 🡫**;** decreased, 🡪; no change, 🡩; increased.

1. Kamemoto A, Ara T, Hattori T, Fujinami Y, Imamura Y, Wang PL. Macrolide antibiotics like azithromycin increase lipopolysaccharide-induced IL-8 production by human gingival fibroblasts. Eur J Med Res. 2009;14(7):309-14. PubMed PMID: 19661014; PubMed Central PMCID: PMCPMC3458641.

2. Gannon SC, Cantley MD, Haynes DR, Hirsch R, Bartold PM. Azithromycin suppresses human osteoclast formation and activity in vitro. J Cell Physiol. 2013;228(5):1098-107. doi: 10.1002/jcp.24259. PubMed PMID: 23065774.

3. Kobayashi Y, Wada H, Rossios C, Takagi D, Higaki M, Mikura S, et al. A novel macrolide solithromycin exerts superior anti-inflammatory effect via NF-kappaB inhibition. J Pharmacol Exp Ther. 2013;345(1):76-84. doi: 10.1124/jpet.112.200733. PubMed PMID: 23359665.

4. Cory TJ, Birket SE, Murphy BS, Mattingly C, Breslow-Deckman JM, Feola DJ. Azithromycin increases in vitro fibronectin production through interactions between macrophages and fibroblasts stimulated with Pseudomonas aeruginosa. J Antimicrob Chemother. 2013;68(4):840-51. doi: 10.1093/jac/dks476. PubMed PMID: 23248239; PubMed Central PMCID: PMCPMC3594493.

5. Ribeiro CM, Hurd H, Wu Y, Martino ME, Jones L, Brighton B, et al. Azithromycin treatment alters gene expression in inflammatory, lipid metabolism, and cell cycle pathways in well-differentiated human airway epithelia. PLoS One. 2009;4(6):e5806. doi: 10.1371/journal.pone.0005806. PubMed PMID: 19503797; PubMed Central PMCID: PMCPMC2688381.

6. Singh S, Kubler A, Singh UK, Singh A, Gardiner H, Prasad R, et al. Antimycobacterial drugs modulate immunopathogenic matrix metalloproteinases in a cellular model of pulmonary tuberculosis. Antimicrob Agents Chemother. 2014;58(8):4657-65. doi: 10.1128/AAC.02141-13. PubMed PMID: 24890593; PubMed Central PMCID: PMCPMC4136059.

7. Verleden SE, Vandooren J, Vos R, Willems S, Dupont LJ, Verleden GM, et al. Azithromycin decreases MMP-9 expression in the airways of lung transplant recipients. Transpl Immunol. 2011;25(2-3):159-62. doi: 10.1016/j.trim.2011.06.006. PubMed PMID: 21740970.

8. O'Reilly PJ, Jackson PL, Wells JM, Dransfield MT, Scanlon PD, Blalock JE. Sputum PGP is reduced by azithromycin treatment in patients with COPD and correlates with exacerbations. BMJ Open. 2013;3(12):e004140. doi: 10.1136/bmjopen-2013-004140. PubMed PMID: 24366582; PubMed Central PMCID: PMCPMC3884851.

9. Fernandez-Robredo P, Recalde S, Moreno-Orduna M, Garcia-Garcia L, Zarranz-Ventura J, Garcia-Layana A. Azithromycin reduces inflammation in a rat model of acute conjunctivitis. Mol Vis. 2013;19:153-65. PubMed PMID: 23378729; PubMed Central PMCID: PMCPMC3559097.

10. Kamat AM, Karashima T, Davis DW, Lashinger L, Bar-Eli M, Millikan R, et al. The proteasome inhibitor bortezomib synergizes with gemcitabine to block the growth of human 253JB-V bladder tumors in vivo. Mol Cancer Ther. 2004;3(3):279-90. PubMed PMID: 15026548.

11. Reikvam H, Hatfield KJ, Oyan AM, Kalland KH, Kittang AO, Bruserud O. Primary human acute myelogenous leukemia cells release matrix metalloproteases and their inhibitors: release profile and pharmacological modulation. Eur J Haematol. 2010;84(3):239-51. doi: 10.1111/j.1600-0609.2009.01382.x. PubMed PMID: 19922462.

12. Cohen K, Flint N, Shalev S, Erez D, Baharal T, Davis PJ, et al. Thyroid hormone regulates adhesion, migration and matrix metalloproteinase 9 activity via alphavbeta3 integrin in myeloma cells. Oncotarget. 2014;5(15):6312-22. doi: 10.18632/oncotarget.2205. PubMed PMID: 25071016; PubMed Central PMCID: PMCPMC4171632.

13. Lee JH, Chiang SY, Nam D, Chung WS, Lee J, Na YS, et al. Capillarisin inhibits constitutive and inducible STAT3 activation through induction of SHP-1 and SHP-2 tyrosine phosphatases. Cancer Lett. 2014;345(1):140-8. doi: 10.1016/j.canlet.2013.12.008. PubMed PMID: 24333736.

14. Tiriveedhi V, Upadhya GA, Busch RA, Gunter KL, Dines JN, Knolhoff BL, et al. Protective role of bortezomib in steatotic liver ischemia/reperfusion injury through abrogation of MMP activation and YKL-40 expression. Transpl Immunol. 2014;30(2-3):93-8. doi: 10.1016/j.trim.2013.12.003. PubMed PMID: 24380732.

15. Dutta K, Mishra MK, Nazmi A, Kumawat KL, Basu A. Minocycline differentially modulates macrophage mediated peripheral immune response following Japanese encephalitis virus infection. Immunobiology. 2010;215(11):884-93. doi: 10.1016/j.imbio.2009.12.003. PubMed PMID: 20153075.

16. Yao JS, Shen F, Young WL, Yang GY. Comparison of doxycycline and minocycline in the inhibition of VEGF-induced smooth muscle cell migration. Neurochem Int. 2007;50(3):524-30. doi: 10.1016/j.neuint.2006.10.008. PubMed PMID: 17145119; PubMed Central PMCID: PMCPMC1876824.

17. Romero-Perez D, Fricovsky E, Yamasaki KG, Griffin M, Barraza-Hidalgo M, Dillmann W, et al. Cardiac uptake of minocycline and mechanisms for in vivo cardioprotection. J Am Coll Cardiol. 2008;52(13):1086-94. doi: 10.1016/j.jacc.2008.06.028. PubMed PMID: 18848143; PubMed Central PMCID: PMCPMC2572824.

18. Ohshima S, Fujimoto S, Petrov A, Nakagami H, Haider N, Zhou J, et al. Effect of an antimicrobial agent on atherosclerotic plaques: assessment of metalloproteinase activity by molecular imaging. J Am Coll Cardiol. 2010;55(12):1240-9. doi: 10.1016/j.jacc.2009.11.056. PubMed PMID: 20298932.

19. Hollborn M, Wiedemann P, Bringmann A, Kohen L. Chemotactic and cytotoxic effects of minocycline on human retinal pigment epithelial cells. Invest Ophthalmol Vis Sci. 2010;51(5):2721-9. doi: 10.1167/iovs.09-4661. PubMed PMID: 20019360.

20. Chen X, Chen S, Jiang Y, Zhu C, Wu A, Ma X, et al. Minocycline reduces oxygen-glucose deprivation-induced PC12 cell cytotoxicity via matrix metalloproteinase-9, integrin beta1 and phosphorylated Akt modulation. Neurol Sci. 2013;34(8):1391-6. doi: 10.1007/s10072-012-1246-z. PubMed PMID: 23224583.

21. Brundula V, Rewcastle NB, Metz LM, Bernard CC, Yong VW. Targeting leukocyte MMPs and transmigration: minocycline as a potential therapy for multiple sclerosis. Brain. 2002;125(Pt 6):1297-308. PubMed PMID: 12023318.

22. Ding JY, Kreipke CW, Schafer P, Schafer S, Speirs SL, Rafols JA. Synapse loss regulated by matrix metalloproteinases in traumatic brain injury is associated with hypoxia inducible factor-1alpha expression. Brain Res. 2009;1268:125-34. doi: 10.1016/j.brainres.2009.02.060. PubMed PMID: 19285046; PubMed Central PMCID: PMCPMC2668731.

23. Higashida T, Kreipke CW, Rafols JA, Peng C, Schafer S, Schafer P, et al. The role of hypoxia-inducible factor-1alpha, aquaporin-4, and matrix metalloproteinase-9 in blood-brain barrier disruption and brain edema after traumatic brain injury. J Neurosurg. 2011;114(1):92-101. doi: 10.3171/2010.6.JNS10207. PubMed PMID: 20617879.

24. Homsi S, Federico F, Croci N, Palmier B, Plotkine M, Marchand-Leroux C, et al. Minocycline effects on cerebral edema: relations with inflammatory and oxidative stress markers following traumatic brain injury in mice. Brain Res. 2009;1291:122-32. doi: 10.1016/j.brainres.2009.07.031. PubMed PMID: 19631631.

25. Garrido-Mesa N, Utrilla P, Comalada M, Zorrilla P, Garrido-Mesa J, Zarzuelo A, et al. The association of minocycline and the probiotic Escherichia coli Nissle 1917 results in an additive beneficial effect in a DSS model of reactivated colitis in mice. Biochem Pharmacol. 2011;82(12):1891-900. doi: 10.1016/j.bcp.2011.09.004. PubMed PMID: 21930116.

26. Rosenberg GA, Estrada EY, Mobashery S. Effect of synthetic matrix metalloproteinase inhibitors on lipopolysaccharide-induced blood-brain barrier opening in rodents: Differences in response based on strains and solvents. Brain Res. 2007;1133(1):186-92. doi: 10.1016/j.brainres.2006.11.041. PubMed PMID: 17184743; PubMed Central PMCID: PMCPMC1861831.

27. Nagel S, Su Y, Horstmann S, Heiland S, Gardner H, Koziol J, et al. Minocycline and hypothermia for reperfusion injury after focal cerebral ischemia in the rat: effects on BBB breakdown and MMP expression in the acute and subacute phase. Brain Res. 2008;1188:198-206. doi: 10.1016/j.brainres.2007.10.052. PubMed PMID: 18031717.

28. Keilhoff G, Schild L, Fansa H. Minocycline protects Schwann cells from ischemia-like injury and promotes axonal outgrowth in bioartificial nerve grafts lacking Wallerian degeneration. Exp Neurol. 2008;212(1):189-200. doi: 10.1016/j.expneurol.2008.03.028. PubMed PMID: 18501894.

29. Mishra MK, Dutta K, Saheb SK, Basu A. Understanding the molecular mechanism of blood-brain barrier damage in an experimental model of Japanese encephalitis: correlation with minocycline administration as a therapeutic agent. Neurochem Int. 2009;55(8):717-23. doi: 10.1016/j.neuint.2009.07.006. PubMed PMID: 19628016.

30. Elgebaly MM, Prakash R, Li W, Ogbi S, Johnson MH, Mezzetti EM, et al. Vascular protection in diabetic stroke: role of matrix metalloprotease-dependent vascular remodeling. J Cereb Blood Flow Metab. 2010;30(12):1928-38. doi: 10.1038/jcbfm.2010.120. PubMed PMID: 20664613; PubMed Central PMCID: PMCPMC3002883.

31. Koistinaho M, Malm TM, Kettunen MI, Goldsteins G, Starckx S, Kauppinen RA, et al. Minocycline protects against permanent cerebral ischemia in wild type but not in matrix metalloprotease-9-deficient mice. J Cereb Blood Flow Metab. 2005;25(4):460-7. doi: 10.1038/sj.jcbfm.9600040. PubMed PMID: 15674236.

32. Machado LS, Kozak A, Ergul A, Hess DC, Borlongan CV, Fagan SC. Delayed minocycline inhibits ischemia-activated matrix metalloproteinases 2 and 9 after experimental stroke. BMC Neurosci. 2006;7:56. doi: 10.1186/1471-2202-7-56. PubMed PMID: 16846501; PubMed Central PMCID: PMCPMC1543649.

33. Maier CM, Hsieh L, Crandall T, Narasimhan P, Chan PH. Evaluating therapeutic targets for reperfusion-related brain hemorrhage. Ann Neurol. 2006;59(6):929-38. doi: 10.1002/ana.20850. PubMed PMID: 16673393.

34. Mathalone N, Lahat N, Rahat MA, Bahar-Shany K, Oron Y, Geyer O. The involvement of matrix metalloproteinases 2 and 9 in rat retinal ischemia. Graefes Arch Clin Exp Ophthalmol. 2007;245(5):725-32. doi: 10.1007/s00417-006-0362-y. PubMed PMID: 17024442.

35. Sutton TA, Kelly KJ, Mang HE, Plotkin Z, Sandoval RM, Dagher PC. Minocycline reduces renal microvascular leakage in a rat model of ischemic renal injury. Am J Physiol Renal Physiol. 2005;288(1):F91-7. doi: 10.1152/ajprenal.00051.2004. PubMed PMID: 15353401.

36. Hoda MN, Li W, Ahmad A, Ogbi S, Zemskova MA, Johnson MH, et al. Sex-independent neuroprotection with minocycline after experimental thromboembolic stroke. Exp Transl Stroke Med. 2011;3(1):16. doi: 10.1186/2040-7378-3-16. PubMed PMID: 22177314; PubMed Central PMCID: PMCPMC3287111.

37. Wang Z, Meng CJ, Shen XM, Shu Z, Ma C, Zhu GQ, et al. Potential contribution of hypoxia-inducible factor-1alpha, aquaporin-4, and matrix metalloproteinase-9 to blood-brain barrier disruption and brain edema after experimental subarachnoid hemorrhage. J Mol Neurosci. 2012;48(1):273-80. doi: 10.1007/s12031-012-9769-6. PubMed PMID: 22528459.

38. Lee CZ, Yao JS, Huang Y, Zhai W, Liu W, Guglielmo BJ, et al. Dose-response effect of tetracyclines on cerebral matrix metalloproteinase-9 after vascular endothelial growth factor hyperstimulation. J Cereb Blood Flow Metab. 2006;26(9):1157-64. doi: 10.1038/sj.jcbfm.9600268. PubMed PMID: 16395286.

39. Matsumoto Y, Park IK, Kohyama K. Matrix metalloproteinase (MMP)-9, but not MMP-2, is involved in the development and progression of C protein-induced myocarditis and subsequent dilated cardiomyopathy. J Immunol. 2009;183(7):4773-81. doi: 10.4049/jimmunol.0900871. PubMed PMID: 19734212.

40. Zhang ZY, Zhang Z, Fauser U, Schluesener HJ. Improved outcome of EAN, an animal model of GBS, through amelioration of peripheral and central inflammation by minocycline. J Cell Mol Med. 2009;13(2):341-51. doi: 10.1111/j.1582-4934.2008.00333.x. PubMed PMID: 18400050; PubMed Central PMCID: PMCPMC3823360.

41. Cayabyab FS, Gowribai K, Walz W. Involvement of matrix metalloproteinases-2 and -9 in the formation of a lacuna-like cerebral cavity. J Neurosci Res. 2013;91(7):920-33. doi: 10.1002/jnr.23223. PubMed PMID: 23606560.

42. Dziembowska M, Pretto DI, Janusz A, Kaczmarek L, Leigh MJ, Gabriel N, et al. High MMP-9 activity levels in fragile X syndrome are lowered by minocycline. Am J Med Genet A. 2013;161A(8):1897-903. doi: 10.1002/ajmg.a.36023. PubMed PMID: 23824974.

43. Bilousova TV, Dansie L, Ngo M, Aye J, Charles JR, Ethell DW, et al. Minocycline promotes dendritic spine maturation and improves behavioural performance in the fragile X mouse model. J Med Genet. 2009;46(2):94-102. doi: 10.1136/jmg.2008.061796. PubMed PMID: 18835858.

44. Bruno MA, Leon WC, Fragoso G, Mushynski WE, Almazan G, Cuello AC. Amyloid beta-induced nerve growth factor dysmetabolism in Alzheimer disease. J Neuropathol Exp Neurol. 2009;68(8):857-69. doi: 10.1097/NEN.0b013e3181aed9e6. PubMed PMID: 19606067.
