# Supplementary material for: Differential inhibition of activity, activation and gene expression of MMP-9 in THP-1 cells by azithromycin and minocycline versus bortezomib: A comparative study
Source: PLoS One. 2017 Apr 3;12(4):e0174853. doi: 10.1371/journal.pone.0174853 (PMC5378356; doi:10.1371/journal.pone.0174853)
Supplement: S1 Protocol — (DOCX) [file pone.0174853.s002.docx]

**S1 Protocol: HUVEC culture, viability and experimental design.**

HUVECs were cultured in flasks coated with 0.2% gelatin and EGM-2MV medium (Lonza, Basel, Switzerland) with 2% FBS. HUVECs (80 000 cells in 2 ml EGM) were seeded in a 0.2% gelatin coated 12-well plate and allowed to settle for 24 hours. Next, growth medium was replaced with serum-free, basal medium (EBM-2) and 50 µl of the test compound was added followed by 50 µl of LPS (final concentration of 10 µg/ml). After 24h (37 ºC and 5% CO_2_) the supernatants were collected and the cells were collected in RNA extraction buffer. HUVEC cell viability was tested with the use of an alamarBlue® cell viability assay (DAL1025 – ThermoFisher Scientific) according to the manufacturer’s instructions. For cell culture medium samples, the total protein content was determined and used to normalize sample preparation before gelatin zymography.
