# Supplementary material for: Differential inhibition of activity, activation and gene expression of MMP-9 in THP-1 cells by azithromycin and minocycline versus bortezomib: A comparative study
Source: PLoS One. 2017 Apr 3;12(4):e0174853. doi: 10.1371/journal.pone.0174853 (PMC5378356; doi:10.1371/journal.pone.0174853)
Supplement: S1 Fig — Cell viability is expressed as the percentage of cells compared to the control condition (LPS only, no compounds). Individual data points are shown and the bars represent the mean value. Data were statistically analyzed using a Bonferroni's multiple comparison test. *, p ≤ 0.05; **, p ≤ 0.01; *** p ≤ 0.001; **** p ≤ 0.0001; n = 3. (DOCX) [file pone.0174853.s003.docx]

**S1 Figure: The effect of minocycline, azithromycin, bortezomib and SB-3CT on cell viability of LPS-stimulated HUVEC cells.**


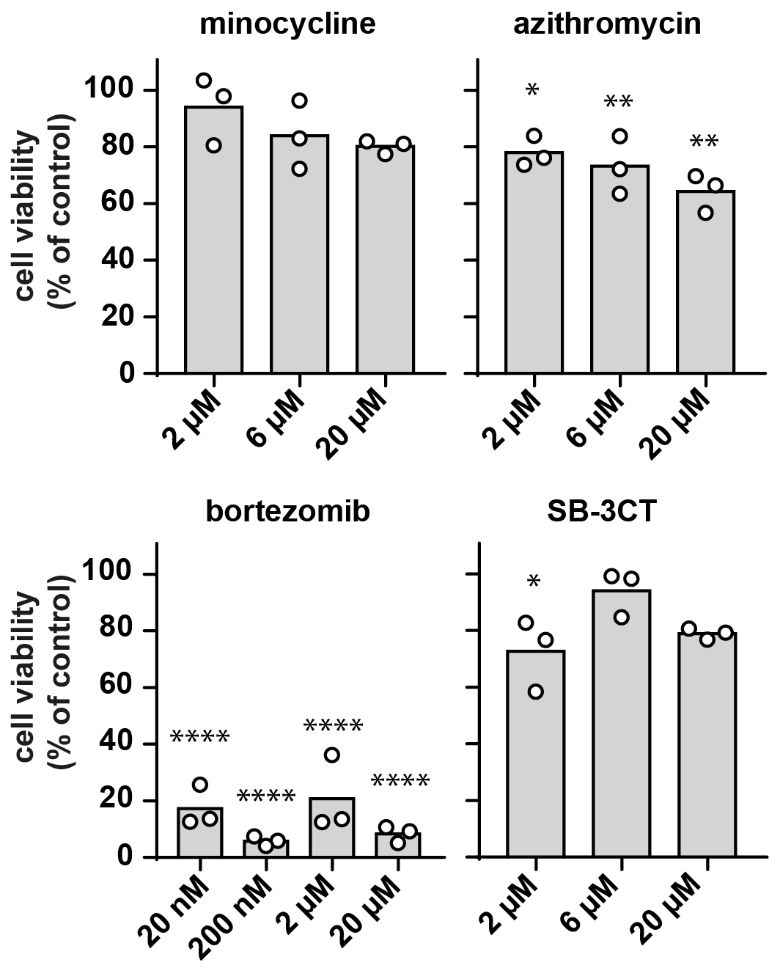


**S1 Fig. The effect of minocycline, azithromycin, bortezomib and SB-3CT on cell viability of LPS-stimulated HUVEC cells.** Cell viability is expressed as the percentage of cells compared to the control condition (LPS only, no compounds). Individual data points are shown and the bars represent the mean value. Data were statistically analyzed using a Bonferroni's multiple comparison test. *, p ≤ 0.05; **, p ≤ 0.01; *** p ≤ 0.001; **** p ≤ 0.0001; n = 3.
