# Supplementary material for: Differential inhibition of activity, activation and gene expression of MMP-9 in THP-1 cells by azithromycin and minocycline versus bortezomib: A comparative study
Source: PLoS One. 2017 Apr 3;12(4):e0174853. doi: 10.1371/journal.pone.0174853 (PMC5378356; doi:10.1371/journal.pone.0174853)
Supplement: S2 Fig — (A) Overview image of a representative zymography gel. To concentrate the sample, cell culture supernatant was first subjected to gelatin affinity chromatography. Loadings are representative for 25 μl cell culture supernatant. MMP-2 appeared as a constitutively expressed gelatinase and no MMP-9 protein was detected. Equal amounts of an internal loading control (±48 kDa MMP-9 mutant lacking the O-glycosylated and hemopexin domains) were included to allow correction for sample processing and loading errors. hrMMP-9, human recombinant proMMP-9. (B) Relative expression of the mRNAs encoded by the human MMP-2, MMP-9 and TIMP-1 genes, as corrected towards that of the housekeeping gene GADPH. While MMP-2 gene expression levels were high and constitutive, MMP-9 expression was insignificant. Compound concentrations were identical to those in panel A. (C) Fold changes of MMP-2 and TIMP-1 mRNA levels, relative to the levels from cells treated with LPS only and corrected towards the housekeeping gene GADPH. Compound concentrations were identical to those in panel A. Individual data points are shown and the bars represent the mean values. Data were statistically analyzed compared to the LPS control using a Bonferroni's multiple comparison test. **, p ≤ 0.01; **** p ≤ 0.0001; n = 3. (DOCX) [file pone.0174853.s004.docx]

**S2 Figure: Zymography and qPCR analysis of HUVEC cells challenged with LPS and treated with minocycline, azithromycin, bortezomib and SB-3CT.**


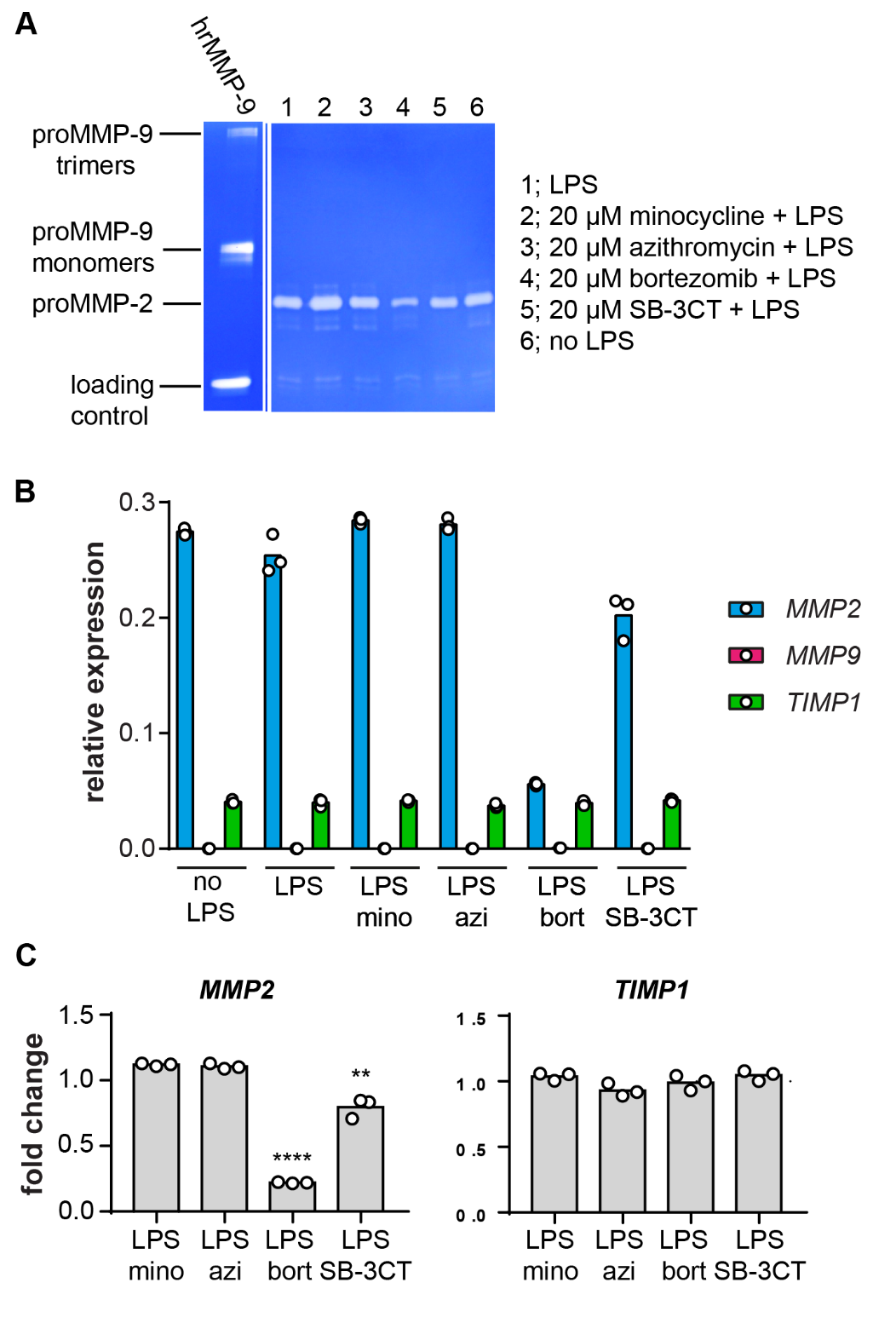


**S2 Fig. Zymography and qPCR analysis of HUVEC cells challenged with LPS and treated with minocycline, azithromycin, bortezomib and SB-3CT.** (A) Overview image of a representative zymography gel. To concentrate the sample, cell culture supernatant was first subjected to gelatin affinity chromatography. Loadings are representative for 25 µl cell culture supernatant. MMP-2 appeared as a constitutively expressed gelatinase and no MMP-9 protein was detected. Equal amounts of an internal loading control (±48 kDa MMP-9 mutant lacking the O-glycosylated and hemopexin domains) were included to allow correction for sample processing and loading errors. hrMMP-9, human recombinant proMMP-9. (B) Relative expression of the mRNAs encoded by the human MMP-2, MMP-9 and TIMP-1 genes, as corrected towards that of the housekeeping gene GADPH. While MMP-2 gene expression levels were high and constitutive, MMP-9 expression was insignificant. Compound concentrations were identical to those in panel A. (C) Fold changes of MMP-2 and TIMP-1 mRNA levels, relative to the levels from cells treated with LPS only and corrected towards the housekeeping gene GADPH. Compound concentrations were identical to those in panel A. Individual data points are shown and the bars represent the mean values. Data were statistically analyzed compared to the LPS control using a Bonferroni's multiple comparison test. **, p ≤ 0.01; **** p ≤ 0.0001; n = 3.
